# Veratrum Viride

**Published:** 1854-05

**Authors:** A. Hard

**Affiliations:** Aurora, Ill.


### *On the Use of Veratrum Viride in Pneumonia.*

BY A. HARD, M. D.

Having just read an article in the Buffalo Med. Journal, signed Rusticus, on the quack preparation of Veratrum Viride, wherein the writer has not limited his remarks to the quack preparation, but has attempted from history to prove the inutility of the drug, I deem it a duty to briefly state what it has accomplished in my hands. Not that I would encourage any person to buy or administer any quack prepation, as any preparation which is offered for sale to the public for their indiscriminate use, let it be prepared by whom it may, should be labelled Quackery.

Veratrum Viride is a therapeutical agent of so great value that I would as soon treat intermittent fever without *Quinine* as Pneumonia without *Veratrum*. In the winter of 1852 and '53, Pneumonia prevailed to an alarming extent, in a certain locality, in La Salle and Grundy Counties, Ill. Nearly all who were attacked with it died.— It created as much fear as Cholera. In April following I was called to several cases in the same neighborhood, which the friends considered nearly hopeless. I treated them all with the saturated tincture of Veratrum Viride. In cases of adults I gave 10 drops every 3 or 4 hours until the pulse was reduced to, or below, the normal standard which was generally accompanied by emesis, profuse perspiration, and faintness, in from 6 to 12 hours. I then reduced the dose to 4 or 5 drops as the case seemed to require. This treatment in connection with the common remedies, laxatives, expectorants, &c., was continued until the patient was convalescent, which was usually within a week after the use of the Veratrum, and in no case was there a relapse nor did any unfavorable symptoms follow. I have used it in every case of that disease which I have treated since and it has never disappointed me in fulfilling the indications for which it was given viz: as an arterial sedative and diaphoretic. I have not hesitated to give it to infants, proportioning the dose to the age, say one drop to a child a year old.

Since writing the above, April 5, 1854, while absent on business, I was called home in haste to see my child, a girl 4 years old. I found her with Pneumonia. Pulse 160 per minute, respiration difficult, sputa rusty colored; she had been sick 7 days and was rapidly failing. In her debilitated state I hesitated to administer Veratrum. However as a *dernier resort* I gave it promptly in 4 drop doses every 4 hours. After the second dose her pulse was reduced to 86 per minute. The dose was then lessened to one drop and continued three days. She had a rapid convalescence.

Aurora, Ill., April 11, 1854.

